# Submerged membrane/adsorption hybrid process in water reclamation and concentrate management—a mini review

**DOI:** 10.1007/s11356-022-23229-9

**Published:** 2022-09-27

**Authors:** Paripurnanda Loganathan, Jaya Kandasamy, Harsha Ratnaweera, Saravanamuthu Vigneswaran

**Affiliations:** 1 Faculty of Engineering, University of Technology Sydney (UTS), P.O. Box 123, Broadway, NSW 2127 Australia; 2 Faculty of Sciences & Technology (RealTek), Norwegian University of Life Sciences, P.O. Box N-1432, Ås, Norway

**Keywords:** Submerged membrane adsorption hybrid system, Adsorption, Water pollutants, Organic micropollutants, Membrane, Water treatment, Activated carbon

## Abstract

Clean water shortage is a major global problem due to escalating demand resulting from increasing human population growth and industrial activities, decreasing freshwater resources and persistent droughts. Recycling and reuse of wastewater by adopting efficient reclamation techniques can help solve this problem. However, wastewater contains a wide range of pollutants, which require removal before it may be reused. Adsorption and membrane processes are two successful treatments used to remove most of these pollutants. Their efficiency increases when these processes are integrated as observed, for example in a submerged membrane adsorption hybrid system (SMAHS). It uses coarse air bubbling/sparging to produce local shear which minimises reversible membrane fouling, improves performance and extends the life of the membrane. Additionally, the adsorbent acts as a buoyant media that produces an extra shearing effect on the membrane surface, reduces membrane resistance and increases flux. In addition, it adsorbs the organics that would otherwise deposit on and cause fouling of the membrane. The use of activated carbon (AC) adsorbent in SMAHS is very effective in removing most pollutants including natural organic matter (NOM) and organic micropollutants (OMPs) from wastewaters and membrane concentrate wastes, the latter being a serious problem in practical applications of the reverse osmosis process. However, certain NOM fractions and OMPs (i.e. hydrophilic and negatively charged ones) are not efficiently removed by AC. Other adsorbents need to be explored for their effective removal.

**Graphical abstract:**

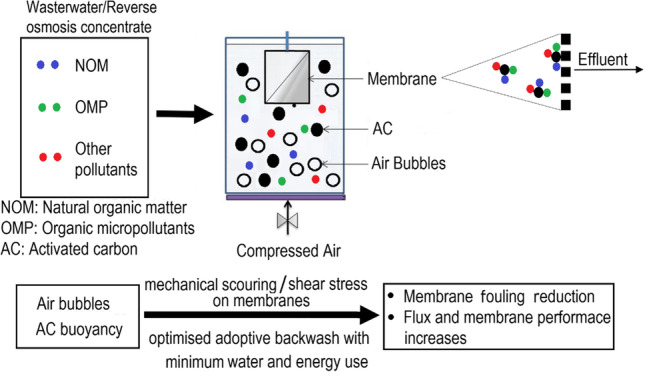

## Introduction

Clean water shortage is a major global problem due to escalating demand resulting from increasing human population growth and industrial activities, decreasing freshwater resources and persistent droughts. Two-thirds of the world’s population is projected to be threatened by a shortage of freshwater by 2025, and climate change is expected to exacerbate this (Zhang and Liu 2021). Yet, enormous quantities of municipal wastewater, industrial water, and stormwater are continuously disposed, at times indiscriminately to the environment. Recycling and reuse of these waters using efficient reclamation techniques can be an effective solution for alleviating the looming water shortage problem. Wastewater generally contains many pollutants such as pathogenic microorganisms, heavy metals, inorganic salts, NOM and OMP (including pharmaceutical and personal care products (PPCPs), endocrine disruptors, pesticides and industrial by-products). If the concentrations of these pollutants are reduced to acceptable levels, the treated water can be beneficially used as non-potable recycled water for irrigating crops, toilet flushing, clothe washing and some industrial activities.

There are several methods of treating wastewater including coagulation/flocculation, activated carbon (AC) adsorption, oxidative and biological processes, membrane-based technologies, electro-dialysis and capacitive deionisation, either alone or in combination (Umar et al. 2015; Arola et al. 2019; Xiang et al. 2019; Valdés et al. 2021; Zhang and Liu 2021). Of these, a combination of methods has generally been found to be more efficient (Xiang et al. 2019; Dewi et al. 2021). This is because the mechanism of removal by each process is different and unique, more suited for removal of a type of pollutant. Therefore, when processes are combined, more diverse mechanisms operate together to enhance the removal of a wider range of pollutants. Pollutants not effectively removed by one process is efficiently removed by the other process. Ejraei et al. (2019) compared several single treatment processes and a combination of them in series for the treatment of pollutants in wastewater. The combined processes were found to perform best, and this was ascribed to the synergetic effects of processes in the hybrid system.

Membrane separation technologies, especially reverse osmosis (RO), is one of the most widely used technology in the world to produce clean water (Joo and Tansel 2015; Yang et al. 2019; Algieri et al. 2021; Valdés et al. 2021; Wu et al. 2021). However, RO generates a waste stream known as RO concentrate (ROC) that generally contains 20–30% of the feed stream (Umar et al. 2015; Yang et al. 2019). ROC contains almost all the pollutants at elevated levels in the feed stream (commonly 2–4 times higher concentration), which may be toxic and/or bio-accumulative (Zhang et al. 2019). Joo and Tansel (2015) reviewed several hybrid systems including membrane processes for the removal of pollutants such as NOM, OMPs, disinfectant by-products and inorganics.

In addition to RO, other membrane processes namely nanofiltration (NF), ultrafiltration (UF) and microfiltration (MF) are now widely used as the principal treatment or as pre-treatment to RO in water treatment plants to remove pollutants. The magnitude of the pore sizes of the membranes used in UF, MF and NF are in the region of 0.1 µm, 0.01 µm and 0.001 µm, respectively (Crittenden et al. 2012). The RO membrane is almost nonporous. A term called molecular weight cutoff (MWCO) is used to designate the smallest molecular weight of a substance that is separated by the membrane with 90% efficiency. The larger the pore size of a membrane the higher the MWCO value. In membrane filtration, the main driving force of water flow through the membrane is the pressure difference over the membrane, commonly referred to as trans-membrane pressure (TMP). TMP is higher for membranes with low MWCO. The removal of pollutants by membranes not only depends on MWCO but also at times on the hydrophobicity/hydrophilicity and electrical charge of the membranes (Jamil et al. 2021a).

MF and UF use membranes of larger pore size and run at lower TMP, require less energy and have lower operating costs. These membranes are referred to as low pressure membranes (LPM) (Stoquart et al. 2012). They effectively remove micro-organisms including pathogens, suspended particle’s turbidity. They are less effective in removing pollutants smaller than the pore size of the membranes such as NOM, OMPs and colour (Lebeau et al. 1998; Matsui et al. 2001). NOM that is not removed causes membrane fouling, which reduces membrane performance and produces irreversible loss in permeate flux, thereby requiring more frequent membrane cleaning and ultimately membrane replacement (Thiruvenkatachari et al. 2004). For these reasons, MF and UF are combined with other processes such as adsorption for more effective application in wastewater treatment plants.

Adsorption is another effective process used in wastewater treatment. This process is cost-effective, simple, eco-friendly, chemical-free, and efficient in removing various inorganic and organic pollutants from a variety of waters and widely used for these reasons. Moreover, it produces minimal amounts of sludge, no undesirable by-products and the adsorbent can be reused. This reduces operation costs. The adsorption process is not only used by itself as a single treatment process, but often employed as post-treatment to enhance the removal of pollutants from wastewater that had escaped removal by oxidation (Margot et al. 2013; Guillossou et al. 2020; Ullberg et al. 2021; Sauter et al. 2021; Loganathan et al. 2022) or coagulation (Choi et al. 2008; Zhang et al. 2019). It is often applied as a polishing step in the production of drinking water (Piai et al. 2020; García et al. 2021).

Recently, there have been increasing applications of the hybrid membrane/adsorption process. Despite this, there have been no systematic reviews of these studies unlike other hybrid treatment systems such as ozonation/adsorption process (Loganathan et al. 2022), ozonation/membrane process (Van Geluwe et al. 2011), biological/electrocoagulation process (Al-Qodah et al. 2020) and coagulation/membrane process (Jiang 2015). This paper reviews the research on using hybrid membrane/adsorption process for removal of various pollutants focusing on NOM and OMPs from different types of wastewaters including ROC. It highlights the advantages of integrating membrane treatment with the adsorption process and the modifications that were made from time to time to improve the design and efficiency of this hybrid process. In recent years, the latest of these designs, SMAHS, is becoming increasingly popular. This process is critically reviewed in the latter part of this paper giving examples of its application to remove different types of pollutants, especially the emerging pollutants such as OMPs and various constituents of NOM.

## Activated carbon (AC) and ion exchange resin adsorbents

Of the various adsorbents, AC is most widely used for removing pollutants having a wide array of adsorption mechanisms that target different pollutants (Löwenberg and Wintgens 2017; Loganathan et al. 2022; Zhang et al. 2022). AC can be produced from any material which has high carbon content, including cheap and abundantly available agricultural wastes (Renu and Singh 2017). AC’s attractiveness is its pollutant removal efficiency stemming from its large surface area (500–1500 m^2^/g), internal porosity, the presence of different types of functional groups, low cost and ready availability (Cougnaud et al. 2005; Pawluk and Fronczyk 2015). Furthermore, the hydrophobic characteristic of the AC enhances the adsorption of many OMPs (Westerhoff et al. 2005; Snyder et al. 2007; Valderrama et al. 2008; Jamil et al. 2019a; Wang et al. 2020) including polycyclic aromatic hydrocarbons (PAHs) (Eeshwarasinghe et al. 2018), colour (Sketchell et al. 1999) and NOM (Velten et al. 2011; Jamil et al. 2021b). The large surface area and presence of pores and channels further enhance AC’s adsorption capacity because pollutants can diffuse into the pores and become adsorbed to its internal surfaces (Eeshwarasinghe et al. 2018). AC, as an adsorbent, has been investigated in numerous studies, either in the form of granulated activated carbon (GAC) or powdered activated carbon (PAC).

AC can also act as a carrier material for biofilm developed from wastewaters, which can biodegrade OMPs and NOM (Seo et al. 1997; Sbardella et al. 2018; Baresel et al. 2019; Piai et al. 2020; Alonso et al. 2021) and provide an additional mechanism for their removal. Piai et al. (2020) demonstrated that used GAC where biofilm had formed generally removed more OMPs. Biologically active GAC removed more OMPs than autoclaved GAC (i.e. without biofilm), because of the microbial degradation of OMPs that occurred in addition to adsorption. Sketchell et al. (1999) developed a biological AC by continuously pumping water through a GAC bed for long periods for a microbial biofilm to develop and then used it in filtration experiments. The high NOM removal observed in the experiments was attributed to the decomposition of the biodegradable NOM by the biofilm that formed on GAC. Sbardella et al. (2018) compared the removal of several OMPs from a wastewater by biologically active AC that had biofilm coatings to biologically inactivated AC in column experiments. Biologically active AC removed 22–35% more OMPs than the inactivated ones.

Ion exchange resins are another type of adsorbents used to remove pollutants, including NOM and OMPs from wastewater. As AC is negatively charged at the pH of natural waters (approximately pH 7), it cannot efficiently adsorb negatively charged pollutants such as humics and some OMPs by electrostatic forces, though other mechanisms such as hydrogen bonding can help its adsorption. The use of positively charged adsorbents helps removal of such negatively charged pollutants.

Khirani et al. (2006) investigated the adsorption of total organic carbon (TOC) from a synthetic wastewater effluent by two PACs and three positively charged anion exchange resins containing quaternary ammonium functional group (Lewatit VP OC1071, Lewatit MP 500 and Purolite A500P). Purolite, when ground to smaller particle size, was found to have approximately the same adsorption capacity as PACs. The effect of grinding was to increase adsorption by creating more charged sites accessible to adsorption. Purolite has a mesoporous structure with more internal adsorption sites available to produce a higher adsorption capacity than the other two resins of microporous gel type. The study found Purolite resin to be a suitable alternative to PAC for removal of NOM present in wastewater.

Shanmuganathan et al. (2014a) compared the removal of various NOM fractions present in biologically treated sewage effluent (BSTE) by an anion exchange resin (Purolite A502PS) and by GAC in fluidized adsorption beds. GAC removed 44% of hydrophobic and 36% of hydrophilic fractions. In addition, 41% of humic hydrophilic fractions were removed. On the other hand, Purolite removed 60% of hydrophobic, 62% of hydrophilic fractions and 67% of the humic hydrophilic fraction. Higher percentage removal of humics by Purolite was due to electrostatic adsorption of the negatively charged humics by the positively charged resin.

Removal by GAC occurs because of hydrogen bonding between humics and surface functional groups in GAC, which also operates in Purolite. Jamil et al. (2019b) studied the removal of NOM constituents present in ROC with batch and column experiments using GAC and the same Purolite (Purolite A502PS) adsorbent media. In both experiments, Purolite completely removed the humics present in the hydrophilic fraction of NOM. GAC only achieved partial removal. A larger NOM removal resulted by combining the two adsorbents sequentially (GAC followed by Purolite) to take advantage of their different adsorbent affinities towards the range of NOM fractions present (Jamil et al. 2020).

## Hybrid membrane/adsorption process for wastewater treatment

The combination of AC adsorption process with MF or UF process is a simple and cost-effective way of removing pollutants. It combines the advantage of the adsorption and biodegradation capability of AC and the effective particle removal ability of membrane filtration. The combined treatment process promises a superior water quality product and improved process stability (Löwenberg and Wintgens 2017).

**Fig. 1:**
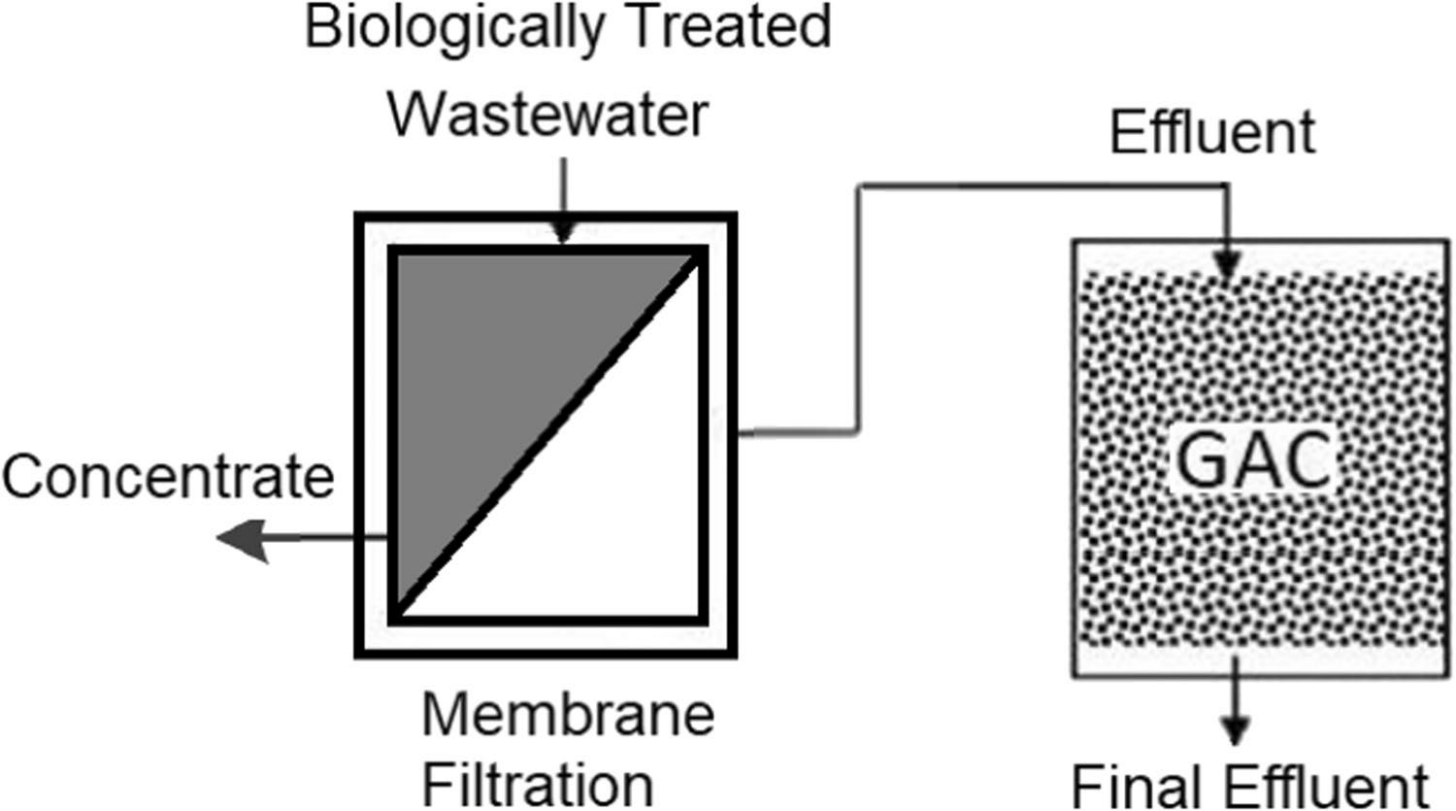
Membrane/adsorption hybrid treatment process with membrane treatment followed by AC adsorption process used to remove wastewater pollutants (modified from Stoquart et al. (2012))

**Fig. 2:**
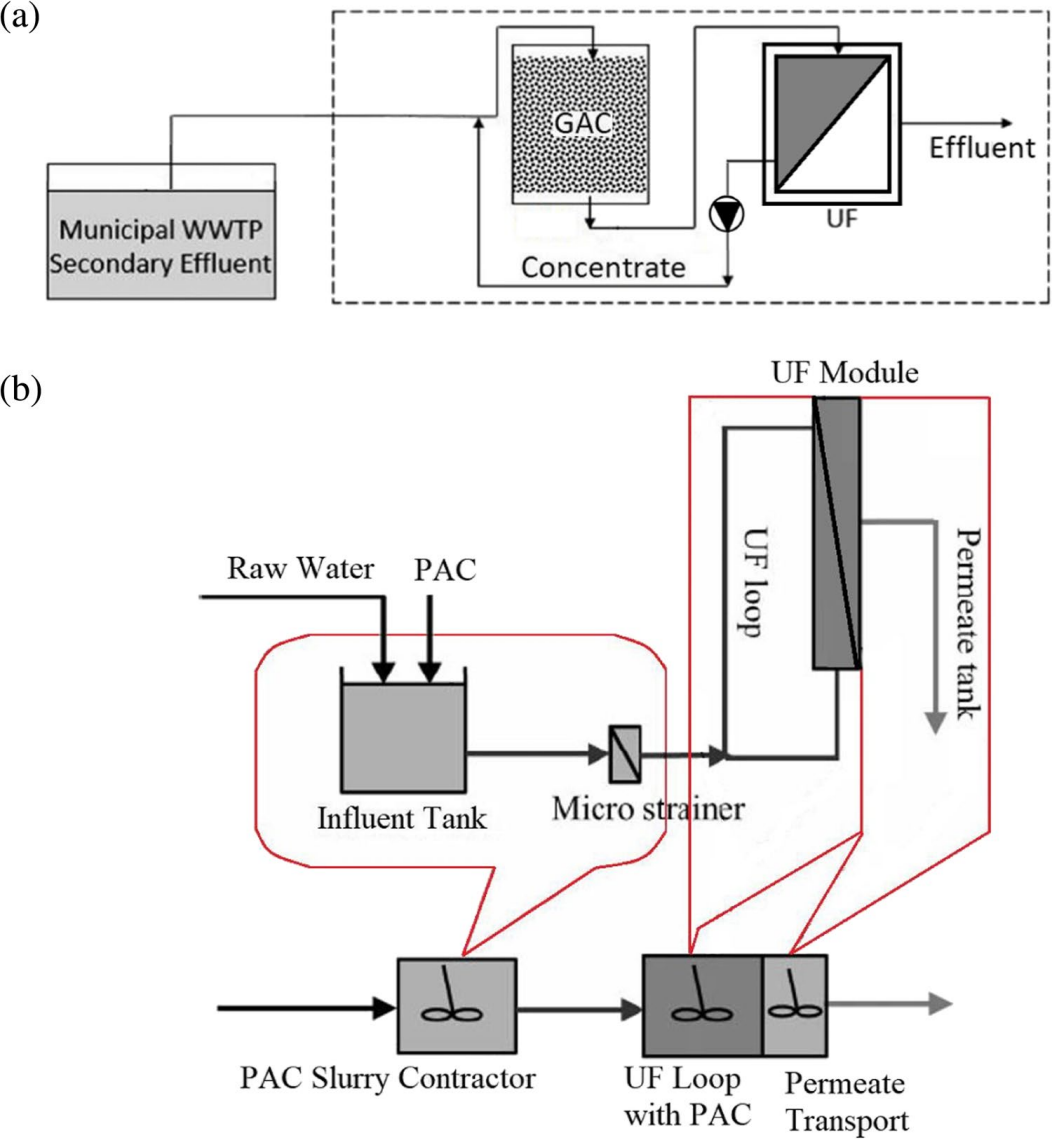
Membrane/adsorption hybrid treatment processes with AC adsorption followed by membrane treatment used to remove wastewater pollutants. (**a**) Feed water passed through GAC column followed by UF (modified from Sbardella et al. (2018)). (**b**) Continuously dosing PAC to the influent feed water followed by UF (modified from Matsui et al. (2001))

**Fig. 3:**
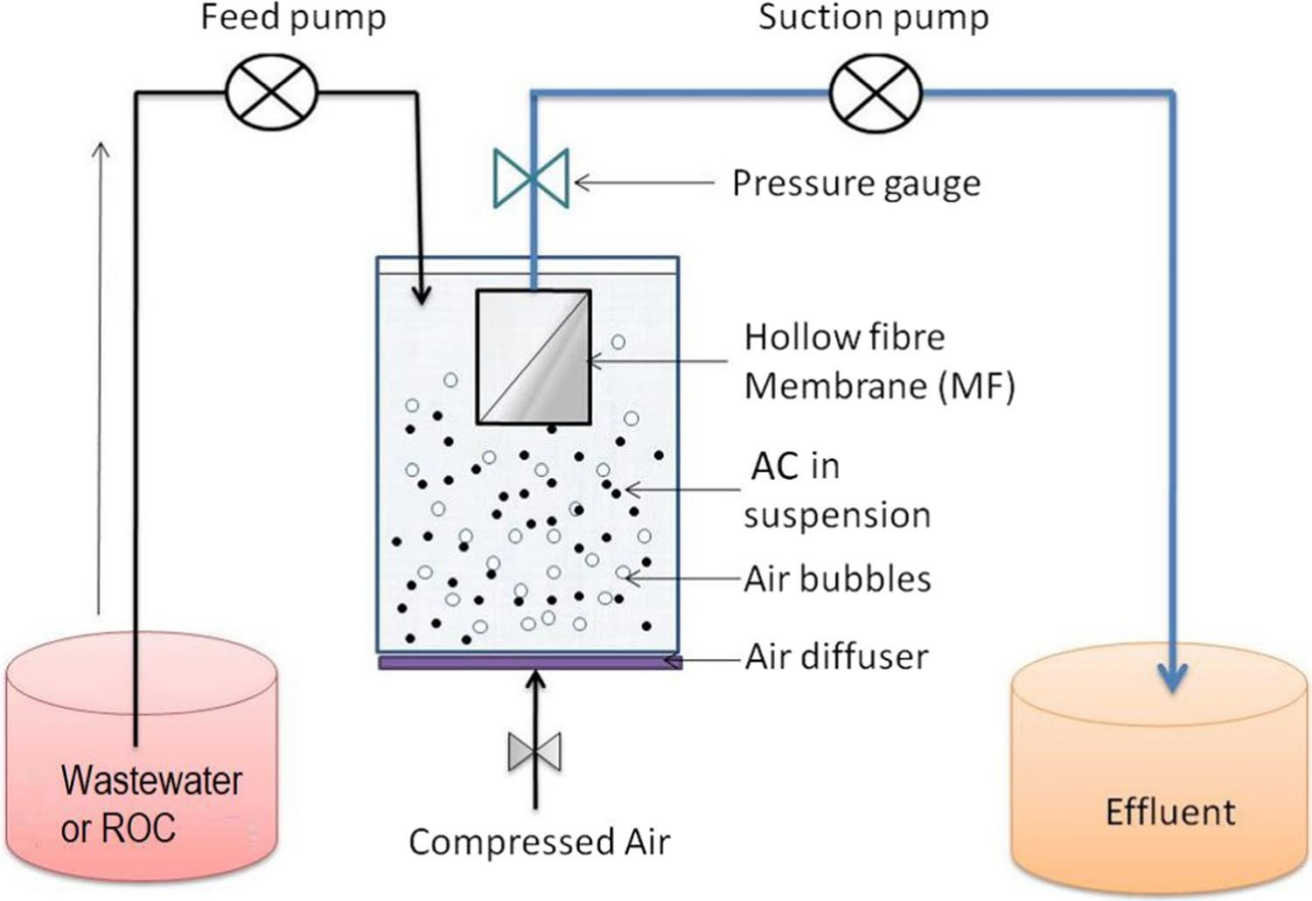
Schematic representation of submerged membrane filtration/AC adsorption hybrid system (modified from Shanmuganathan et al. (2017))

There can be three types of AC/membrane hybrid system design: membrane filtration followed by AC adsorption, AC adsorption followed by membrane filtration and the two processes operating together in a single reaction tank. These configurations are illustrated in Figs. 1, 2 and 3, respectively. When membrane filtration is used first before AC adsorption, particles larger than the membrane pore size are effectively removed from the wastewater, including micro-plastics, bacteria and pathogens. This resulted in a significant reduction of clogging and backwash frequency of the subsequent AC fixed-bed filter, improving the operating conditions of AC. The result for this type of system was reported in a long-term pilot-plant study using wastewater sourced from a treatment plant using UF and GAC biofilter. OMPs, micro-plastics and bacteria present in the water were removed by the hybrid system to below detection limits or to very low concentrations (> 90–98% removal) (Baresel et al. 2019). A problem with using AC after membrane filtration is that some of the AC fines could become exported along with the treated water requiring a subsequent physical separation post-treatment process (Stoquart et al. 2012).

Compared to using membrane filtration followed by AC adsorption, there are more studies reporting AC adsorption followed by membrane filtration. The latter system has long been in operation in many treatment plants. The main reason for having adsorption pretreatment preceding the membrane process is to adsorb the NOM that would otherwise pass through the large pores of the membrane to the treated water (Snoeyink et al. 2000; Thiruvenkatachari et al. 2004; Vigneswaran et al. 2007). The membrane then separates out the organics laden PAC (Vigneswaran et al. 2007). By adsorbing NOM, PAC reduces membrane fouling by removing a major fouling agent thereby prolonging the membrane lifetime (Lin et al. 1999; Shanmuganathan et al. 2017; Lou et al. 2020) and increasing the permeate flux (Vigneswaran et al. 2003). Reducing membrane fouling helps mitigate the rise in TMP and so decreases the operational energy required for filtration (Pan et al. 2022).

There are basically two methods to employ AC as pretreatment to the membrane process. They are: (1). Continuously passing feed water through a GAC column and its effluent fed to membrane system (Zhang et al. 2015; Sbardella et al. 2018; Jamil et al. 2021b) (Fig. 2a); (2). Continuously dosing PAC into the influent feed water, backwashing the used PAC waste that accumulates on the membrane surface, and returning the backwash to the MF/UF module (UF loop in Fig. 2b) (Snoeyink et al. 2000; Matsui et al. 2001)).

Both Fig. 2a and b show the pretreatment option where the wastewater firstly passes through the GAC filter and PAC contactor, respectively. The removal of contaminants takes place prior to membrane filtration. Removing major fouling agents, especially organics prolongs the membrane life and increases the permeate flux. The effluent after the GAC filter or carbon contactor is passed through the membrane. The first configuration is easy to operate and is used in practice. The GAC filter requires periodic backwash to avoid becoming blocked by suspended solids present in the wastewater. The GAC needs to be replaced/regenerated once its adsorptive capacity is exhausted, although this operation is performed after for a long period of time (once a year, for example). Continuous use of GAC can lead to microbial growth on the GAC surface which can degrade the adsorbed organics prolonging its effectiveness in contaminant removal (Alonso et al. 2021). The second configuration (Fig. 2b) uses PAC. The adsorptive capacity and adsorption kinetic of PAC are higher due to its large surface area/specific surface. In this system, optimisation of removal efficiency is possible by varying the dose of PAC and contact time. However, the membrane surface can become damaged due to abrasion as PAC brushes past its surface potentially leading to more frequent membrane replacement. Regeneration of PAC is not economically viable and so the PAC slurry after few passes over the membranes (or after its adsorptive capacity becomes exhausted) needs to be disposed. Although the viability of the second configuration is scientifically proven through extensive research and pilot studies, it is yet to be used in practice.

## Submerged membrane adsorption hybrid system

Compared to having AC adsorption and membrane filtration as separate treatment systems, combining the two treatments in a single tank has many advantages (Vigneswaran et al. 2007; Shanmuganathan et al. 2015a, b, 2017; Pan et al. 2022) (Fig. 3). In the SMAHS hybrid system, the AC or ion exchanger such as Purolite is directly added into the membrane reactor tank where the membrane is submerged in the influent. Adsorption and membrane separation take place simultaneously in a single influent tank. An air diffuser provides a constant airflow into the AC suspension, and the air bubbles induce shear stress and favourable hydraulic distribution over the entire surface of the membrane. In addition, the air flow suspends the AC particles in the reactor tank.

Clogging and channeling problems frequently observed in AC column adsorption (Fig. 2a) do not occur when AC particles are held in suspension (Fig. 2b). The adsorbents in the suspension reduce membrane fouling by adsorbing potential organic foulants before they reach the membrane surface and by mechanically scouring the membrane surface preventing a build-up of TMP. These effects minimise the frequency of membrane cleaning and extend its operational life. These advantages have increased the popularity in recent times of the combined hybrid treatment system for the removal of NOM, OMPs and inorganic pollutants from wastewater. In the SMAHS, a large amount of AC is added only at the commencement of the treatment process (e.g. 5–10 g/L of AC) followed by a daily periodic substitution of only 2–5% of AC which corresponds to an average AC residence time of 20–50 days in the tank. This helps to economise the use of AC without exhausting it.

Management of membranes fouling and controlling it while operating is a major challenge to the widespread application of SMAHS in water reclamation. Membrane scouring with coarse air bubbles (air bubbling/sparging) is one of the most efficient means of minimising reversible fouling and sustainable operation (Pradhan et al. 2012). Coarse air bubbles traversing across the surface of the membrane creates local shear stresses, which minimises membrane fouling and maintains good hydraulic distribution in the membrane system. Bacteria, soluble compounds and membrane material can still interact and prevent control of membrane fouling by aeration alone (Johir et al. 2011). Incorporating supporting media/adsorbents in the membrane tank is a useful method to scrub out many of the foulants that deposit on the surface of the membrane and remove some of the substances that cause fouling before they firmly attach onto the membrane surface (Aryal et al. 2010; Johir et al. 2011; Pradhan et al. 2012). A submerged MF process used to treat a kaolin clay suspension demonstrated how the flux declined with time and attenuation rate increased at higher permeate flux (Aryal et al. 2010). TMP development was higher at higher flux. The introduction of anthracite (640–2000 µm) functioning as a buoyant/supporting medium into the suspension in the membrane tank reduced the flux decline and the rise of TMP by two- to threefold.

Johir et al. (2011) conducted a detailed SMAHS study with biologically treated wastewater effluent on how suspended support media reduced fouling of the membrane. They compared fouling produced in the membrane (0.14 µm) of the SMAHS with and without the addition of GAC (300–600 µm) as a suspended medium (0.5–2 g/L of volume of reactor) at different filtration flux (5–30 L/m^2^ h) and aeration rate (0.5–1.5 m^3^/m^2^ membrane area/h). TMP rose suddenly when the aeration rate reduced from 1.5 to 1.0 m^3^/m^2^ membrane area/h. An addition of GAC prevented this sudden rise of TMP (i.e. reduced membrane resistance) by scouring of the membrane surface which reduced particle deposition on it, and by the additional shearing effect on to membrane surface. Membrane fouling had also reduced. The authors concluded that suspended media, in amounts that depended on the flux and aeration rate used, could effectively reduce membrane fouling. A molecular weight distribution (MWD) and excitation emission matrix (EEM) analysis of the effluent of the bioreactor showed that GAC removed a range of organics (amino acids, biopolymers, humics and fulvic acids type substances) by adsorption mechanisms in addition to the scouring effect that reduces the deposition of particles on to membrane surface.

The above-mentioned study was extended to investigate the effect on membrane fouling by a combination of air flow (sparging/air scouring) together with the use a support medium (GAC, 300–600-µm diameter, dose 1 g/L) with a kaolinite suspension (Pradhan et al. 2012). Membrane fouling was low for high air scouring rates. The presence of support medium further reduced particle deposition on the membrane surface. A doubling of the air flow rate (from 600 to 1200 L/h/m^2^), without granular medium, reduced TMP development by 32% at 10 L/h/m^2^. A similar result (31% reduction) was obtained at the lower air flow rate of 600 L/h/m^2^ with the addition of the granular medium. The study concluded that an addition of support medium together with air flow is a good alternative to a very high air flow in submerged membrane microfiltration systems. Less energy intensive operations can be designed by optimising for both the effects of support medium and air flow. The addition of support medium that are also adsorbents (e.g. GAC) provides additional benefits of removing organics and OMPs as well as adsorbing foulants. These aspects are discussed in detail in the next section.

A detailed hydrodynamic study on submerged flat sheet microfilter system was conducted to determine the effect of air flow rate on deposition on membranes (Pradhan et al. 2014). A concentrated suspension of kaolin clay (10 g/L) was used in an aerated tank containing submerged membranes. This concentration was in a range similar to biomass concentration in SMBR systems. The effect of operating conditions, such as filtration flux, air flow rate, volume of filtered water per unit of membrane area, membrane resistance and particle deposition, was studied. Cake resistance was the major resistance contributing to total membrane resistance. At a flux rate of 15 L/m^2^/h, particle deposition reduced by almost 60% when the air flow rate was tripled from 600 to 1800 L/m^2^/h of membrane area. Particle deposition and TMP rose with higher permeate flux. However, both were reduced by increased air flow rates. It was found that the cake resistance is proportional to the multiplication of flux and cake deposition. The effect of air flow on cake porosity was observed to be more significant at low permeate flux rate.

Although organic fouling on the membrane can be minimised by addition of support medium and air flow, it cannot be totally mitigated. A periodic back flush during the membrane filtration process is required to satisfactorily remove most of the reversible fouling that results in reduced pressure drop and permeate flux decline, ensuing for a longer operation period before it is terminated for physical and/or chemical cleaning of the membrane (Smith et al. 2005, 2006). For successful long-term operation of the membrane process, the optimum frequency and duration of the backwash are necessary. Too short a backwash duration does not completely remove the reversible component of the foulant layer, while too long a duration, though completely removing all the reversible component of the foulant layer, consumes too large a volume of permeate for backwash, reducing the productivity of the system and raising the energy requirement (Smith and Vigneswaran 2009).

Automated system and a supervisory control and data acquisition (SCADA) system together with adaptive backwash initiation and duration schemes were used in a pilot study conducted in a sewage treatment plant in Sydney, Australia. It was found to be very useful and cost effective in the optimal operation of a membrane adsorption hybrid system (Smith and Vigneswaran 2009). Such an approach was used in the development of the automation and SCADA systems, leading to the development of two new control systems for back wash.

The first system involved a closed loop control of the initiation of backwash, based on the gradient of TMP increase. This led to productivity improvements as the backwash is only activated when required, not at a periodic time interval (Smith et al. 2005). This system resulted in a 40% reduction in the water required for backwash and, also, enabled optimised operations under unsteady concentrations of influent wastewater (Smith et al. 2006). In sewage treatment plants, water quality is not static and changes with time. The second system involved an automatic control of the backwash duration, whereby the backwash was terminated when the increase in pressure reached a steady state (or plateau) (Smith et al. 2006). It resulted in a further reduction of water used for backwash of up to 25%.

Periodic replacement of 1.5% of the PAC slurry mixture (i.e. 15 g of PAC replacement/d) was found to have a positive impact on the reduction of membrane fouling. The amount of initial addition of PAC was 1 kg/m^3^ water in the reactor. The membrane fibres were cleaner and freer of slime and solids that form on it when small amounts of PAC are replaced periodically from the membrane reactor (Smith and Vigneswaran 2009). In this study on the treatment of secondary treated sewage effluent, the optimal occurred where in each cycle backwashing was triggered when TMP reached 3 kPa. Frequent backwashing such as this prevented fouling from becoming significant and prevented formation of irreversible cake layers. This allowed the backwashing to occur as often as required yet avoiding too frequent backwashing. The latter results in a decline in the productivity of the system.

## SMAHS in water and wastewater treatment

Intensive studies on the application of SMAHS in water and wastewater treatment commenced in the late 1990s to 2000s, and useful information was obtained on improving the operation conditions of SMAH for achieving successful removal of water contaminants (Vigneswaran et al. 2003; Guo et al. 2005; Kim et al. 2005; Ji and Zhou 2006; Saravia et al. 2006). Table 1 presents the major findings achieved from selected important studies.

**Table 1:** Major research findings on SMAHS applications to wastewater treatment (research findings on SMAHS applications in reverse osmosis concentrate treatment are presented in “SMAHS for removing pollutants from reverse osmosis concentrate” section of the paper)

Water	Adsorbent	Membrane	Research findings	Reference
Synthetic wastewater	PAC	MF	With no PAC pre-adsorption, TOC removal was slightly more than with PAC pre-adsorption, and membrane fouling was reduced (TMP reduced). Higher aeration rate increased TOC removal. Backwash reduced TMP development. Increase PAC dose did not affect TOC removal much although TMP reduced. Lower filtration flux led to higher TOC removal and lower TMP	Guo et al. (2005)
Synthetic wastewater	PAC	MF	One month operation. PAC dose 0, 10, 40 g/L. TOC adsorption on membrane (mg/m^2^): 380, 280, 64. Fastest TMP rise was for 0 g/L and slowest for 40 g/L. TOC removal 51–83%, UV254 removal 63–95%	Kim et al. (2005)
Synthetic wastewater	GAC	MF, flat sheets 0.14-µm pores	GAC addition (150–300, 300–600, 600–1200 µm) achieved an additional 10% DOC and COD removal. Highest removal for 150–300 µm. TMP was highest with no GAC, lowest for 300–600 µm. DOC removals for four fractions determined. Humics fraction had the largest percentage removal	Johir et al. (2013)
Kaolin suspension		MF flat sheets 0.14 µm pores	Adding GAC in the suspension (mechanical scouring) with air flow (air scouring) could be a sustainable alternative to applying high air flow rate in SMAHS. Provides additive effect	Pradhan et al. (2012)
Synthetic wastewater	PAC	Hollow fibre membr 0.1 µm	A new control system was developed to automatically optimise the backwash duration and frequency in SMAHS and was tested it in an experiment with a SMAHS. The new system was found to reduce more than 40% of the permeate required for back flushing	Smith et al. (2005)
BTSE**	GAC	MF and NF	The dual membrane hybrid system (MF–GAC followed by NF) was found more effective than MF-GAC system in removing organics, PPCPs and inorganics (Ca^2+^, Mg^2+^, SO_4_^2−^). Removal percentages of DOC fractions were compared	Shanmuga-nathan et al. (2015a)
BTSE**	Purolite A502PS	MF	0.5 g/L Purolite (a dose much lower than for common AC) increased organic removal from less than 10% to above 40%. A higher dose led to better organic removal and reduced membrane resistance. An increase in membrane flux led to higher organics adsorption on the membrane increasing TMP. Increased Purolite particle size reduced DOC removal and TMP development	Shanmuga-nathan et al. (2014b)
Synthetic wastewater	Fe hydroxide, oxyhydroxide	UF	70–80% phosphate removal from 2 mg P/L solution using 0.25–0.60 mg Fe/L of adsorbents. Parameters affecting the performance of the SMAHS were evaluated	Hilbrandt et al. (2019)
Synthetic seawater	KCoFC***, AMP***	MF	When KCoFC (0.2 g/L) was added to 4 L solution (5 mg Rubidium (Rb)/L) and a further 25% of the initial dose added every hour, Rb removal was steady at 90–96% for 26 h. AMP removal was 80–82%	Nur et al. (2018)

**Biologically treated sewage effluent; ***Potassium cobalt hexacyanoferrate, ammonium molybdophosphate

Lebeau et al. (1998) successfully used SMAHS to treat river water. Here, MF membrane and PAC were directly immersed in a tank receiving raw river water. The SMAHS system was tested for removal of NOM, atrazine, microorganisms and turbidity in bench and pilot scale (running for 1 year) experiments. The authors observed that all these pollutants were effectively removed unlike earlier studies where these could not be removed when only the membrane process was used. Aeration was provided to the SMAHS to create turbulence in the vicinity of membrane and to avoid PAC sedimentation within the reactive tank. However, TMP increased slightly during the experiments showing progressive fouling of membrane.

During this period, the emphasis was on removing organics from water not only because they cause problems such as colour, taste and odour and act as substrate for bacterial growth and formation of disinfectant by-products, but also because they cause membrane fouling/clogging and decrease in permeate flux. In all those studies, PAC was used as the adsorbent, and MF membrane was the most commonly used membrane. In SMAHS, PAC adsorbed the organics, and eventually the membrane separates out the organic-laden PAC (Vigneswaran et al. 2007). More importantly the low molecular weight hydrophilic NOM fractions which can pass through the membranes are adsorbed by PAC (Uyak et al. 2014). More adsorption sites become available on the PAC surface as organics that were adsorbed biodegrade (Vigneswaran et al. 2007). The submerged membranes do not experience clogging because the PAC removes virtually all organics. The PAC is separated out by the membrane.

Increase in PAC dose and aeration rate enhanced organics removal but higher filtration flux reduced organics removal (Vigneswaran et al. 2003; Thiruvenkatachari et al. 2004; Guo et al. 2005; Kim et al. 2005). TMP development reduced with larger doses of PAC and lower filtration flux (Guo et al. 2005). The reduction in TMP was helpful in preventing membrane clogging. Kim et al. (2005) reported TOC removal of 51, 74 and 83% from synthetic wastewater for PAC doses of 0, 10 and 40 g/L, respectively. It should be noted that PAC was added only at the beginning of the experiment. The corresponding amounts of organic compounds that attached to the membrane pores decreased to 380, 280 and 64 mg of TOC per 1 m^2^ of membrane surface area, respectively.

**Fig. 4:**
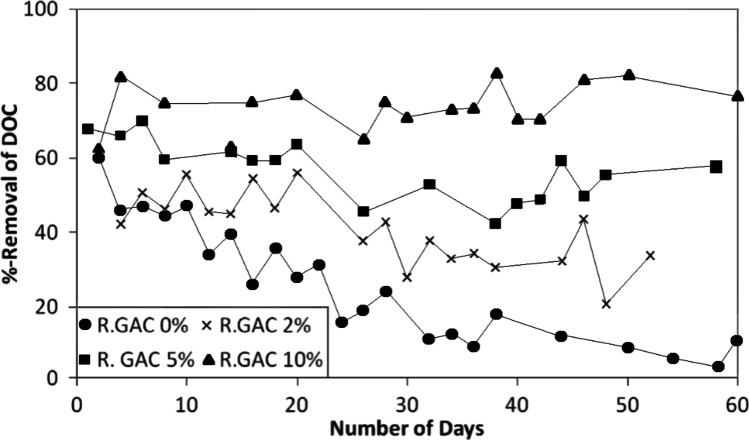
Effect of increasing rate of GAC replacement on DOC removal from BTSE using SMAHS (DOC of initial BTSE 4–7 mg/L; Initial GAC dose 2 g/L; R.GAC denotes daily replacement rate of GAC) (modified from Shanmuganathan et al. (2015a))

In the SMAHS, it is necessary to steadily renew the aged PAC because the adsorption capacity of PAC continues to decline with time due to the progressive amounts of organics being adsorbed leaving less sites for further adsorption. It is possible that some of the adsorbed pollutants can desorb when the solution concentration becomes low. Therefore, it is important to replace the exhausted adsorbent before desorption of the adsorbed pollutants commences (Pan et al. 2022). Shanmuganathan et al. (2015a) conducted a number of SMAHS experiments on organic removal using biologically treated sewage effluent (BTSE). The initial GAC dose that was added for all experiments was 2 g/L, and the daily replacement doses were 0%, 2%, 5% and 10% GAC. They reported that a daily replacement dose of 5% and 10% GAC kept DOC removal steady at between 40 and 70% and 60 and 80% respectively from the commencement of the experiments for a period of 60 days (Fig. 4) when the experiment was stopped. At the lower replacement dose of 2% GAC, the removal decreased from approximately 50 to 20% during this period. Although with renewing, PAC has high capacity to absorb the organic matters, too frequent replacement can lead to low growth of microorganisms and hence a reduction in biodegradation of organics (Jeong et al. 2013).

A simple mathematical model based on CSTR (continually stirred tank reactor) was developed to predict total organic carbon (TOC) in SMAHS effluent (Vigneswaran et al. 2003). The model accounts for PAC adsorption of organics, membrane separation of organic laden PAC and organic adsorption on the membrane surface. Membrane correlation coefficient (MCC) is a term used in the model that groups membrane adsorption of organics and membrane separation of PAC. The principal parameters found by the model to control the system’s effluent quality were the MCC and the filtration flux. A larger value of MCC gave a better removal of organics by the system. The MCC value was proportional to the dose of PAC added to the system. The hybrid system performance was successfully modelled for the PAC dose in the range of 100–1000 mg/L.

The pollutants’ removal efficiencies are similar when GAC was used instead of PAC in SMAHS in later years (Kim et al. 2009; Johir et al. 2011, 2013; Shanmuganathan et al. 2015a, b). Kim et al. (2009) reported that UV260 removal efficiency of SMAHS using MF and GAC was about 60% whereas that by MF alone was 30%. The rate at which the permeability of the membrane decreases was smaller than a conventional MF membrane process (e.g. 130 days with GAC and about 70 days without GAC). This was attributed to the lower organic loading on the membrane that results from NOM adsorption on the GAC. Treatment with SMAHS gave removal efficiencies for DOC, chemical oxygen demand, total N and total P of 42%, 53%, 15% and 13% respectively, while treatment with MF membrane-alone was 25–30%, 20–25%, 5–10% and 5–8%.

Shanmuganathan et al. (2014b) reported that a smaller particle size of adsorbent gave rise to rapid TMP development in the membrane hybrid system even though removal of DOC was more efficient. Johir et al. (2013) studied more closely the effect of different particle sizes of GAC. It was found that membrane fouling was more effectively reduced by 300–600-µm size GAC than 150–300-µm or 600–1200-µm size GAC. The TMP development was high (38.9 kPa) if no GAC was added. The TMP development was 21.3, 16.0 and 28.5 kPa with GAC sizes of 150–300, 300–600 and 600–1200 µm, respectively. The lowest development of TMP with the use of the 300–600-µm particle size was the result of the combined effect of higher amount of adsorption of organic matter when a larger particle size was used and the greater mechanical scour on the membrane surface created than when using a smaller particle size.

During the last decade, several OMPs consisting mainly of PPCPs, endocrine disruptors, insecticides and industrial by-products have emerged as water pollutants of serious concern (Loganathan et al. 2022). OMPs’ exposure is known to elevate carcinogenic, mutagenic and reproductive toxicity risks in human and animal (Fick et al. 2010; Pal et al. 2010; Cizmas et al. 2015; Zhang et al. 2022). This has led to the application of SMAHS in removing these pollutants from wastewaters. Also, during this period, the emphasis shifted to studying the relative efficiencies of the different NOM fractions’ removal, whereas previously only total NOM removal was studied. This has helped to understand which NOM fraction caused fouling problems and the steps to arrest them. There can also be competition for adsorption between the different NOM fractions and OMPs which reduces the efficiency of removal of these pollutants.

AC is a popular adsorbent that has been used for the efficient removal of NOM and majority of MOPs from wastewater (Snyder et al. 2007; Velten et al. 2011; Jamil et al. 2019a; b; Zhang et al. 2022). Therefore, when AC is used in SMAHS, it can effectively remove NOM and MOPs and stop them from passing through the membranes.

Also, the fouling caused by NOM can be reduced because most of the NOM particles, which would otherwise deposit on the surface and in pores of the membrane, are removed by AC adsorption. Shanmuganathan et al. (2015a) demonstrated how effective MF-GAC hybrid systems were in removing the major organic foulants from BSTE. Both the hydrophobic and hydrophilic forms of NOM were removed, and this led to reduced membrane fouling.

Hydrophobics were adsorbed due to interaction with the hydrophobic GAC surface and *π*-*π* bonding (Loganathan et al. 2022). On the other hand, GAC removal of hydrophilic compounds was governed by mechanisms which are independent of hydrophobicity such as surface complexation, anion exchange and hydrogen bonding. Because they are smaller in size than hydrophobics they can penetrate the pores/channels of the AC and become adsorbed.

Löwenberg et al. (2014) reported that on an average 71%, 56% and 37% of biopolymers, low molecular weight substances and humic substances, respectively were removed from a waste treatment plant effluent using a PAC/UF-based SMAHS (PAC dose 20 mg/L) over a period of 6 months. They also stated that five OMPs in the effluent were effectively removed, and the percentage removal was positively correlated with the log D values of OMPs (a measure of hydrophobility), as reported by Snyder et al. (2007) for eight OMPs adsorbed on GAC (where log Kow was used as a measure of hydrophobicity). The OMPs, benzotrizole and carbamazepine with the highest percentage removal had relatively high log *D* values of 1.44 and 2.45, respectively. The lowest percentage removal was found for sulfamethoxazole which had the smallest log *D* of − 1.51 (Löwenberg et al. 2014). The charge of the OMP also influences its adsorption on AC (Jamil et al. 2019a); negatively charged OMPs are less favourable for adsorption on the negatively charged AC because of electrostatic repulsion compared to positively or neutrally charged ones. For example, Jamil et al. (2019a) found that atenolol (log kow 0.16, positively charged), paracetamol (log kow 0.46, neutral charge) and trimethoprine (log kow 0.91, neutral charge) although having hydrophilic characteristics (low log kow), were almost completely removed (90–100%) by a GAC.

SMAHS has also been shown to effectively remove specialised OMPs such as phenol and its derivatives from industrial wastewaters using adsorbents such as hyper cross-linked polymer adsorbents instead of AC (Ipek et al. 2012). Nguyen et al. (2021) reported that continuous use of MF/PAC-based SMAHS effectively removed nonylphenol ethoxylate from an industrial water.

## SMAHS for removing pollutants from reverse osmosis concentrate

ROC is a wastewater generated in wastewater treatment plants from the RO process. ROC contains high concentrations of salinity, nutrients (phosphorus, nitrogen), NOM and OMPs. Adverse effect on the ecology and environment can result with indiscriminate disposal of partially or untreated ROC (Umar et al. 2015; Joo and Tansel 2015; Valdés et al. 2021; Arola et al. 2019; Zhang and Liu 2021) as well as loss of a scarce water resource (20–30% of total wastewater volume (Umar et al. 2015; Yang et al. 2019)). In many jurisdictions, sustainable treatment and management and safe disposal of ROC are mandated. Removal of pollutants from ROC to comply with the recommended effluent discharge concentration limits is generally more difficult with ROC than wastewater because the pollutant concentrations are so much higher in ROC. For example, the DOC concentration in RO feed or BTSE is in the range of 3–7 mg/L, whereas this concentration in ROC is between 13 and 57 mg/L (Shanmuganathan et al. 2015b, 2017; Jamil et al. 2019a, b; Zhang and Liu 2021).

Hybrid membrane/AC adsorption process has not been widely applied to remove pollutants in ROC. However, the few studies conducted have shown how effective this method is in removing organic pollutants such as NOM and OMPs. These studies are reviewed below, and suggestions are made for improving this process.

**Fig. 5:**
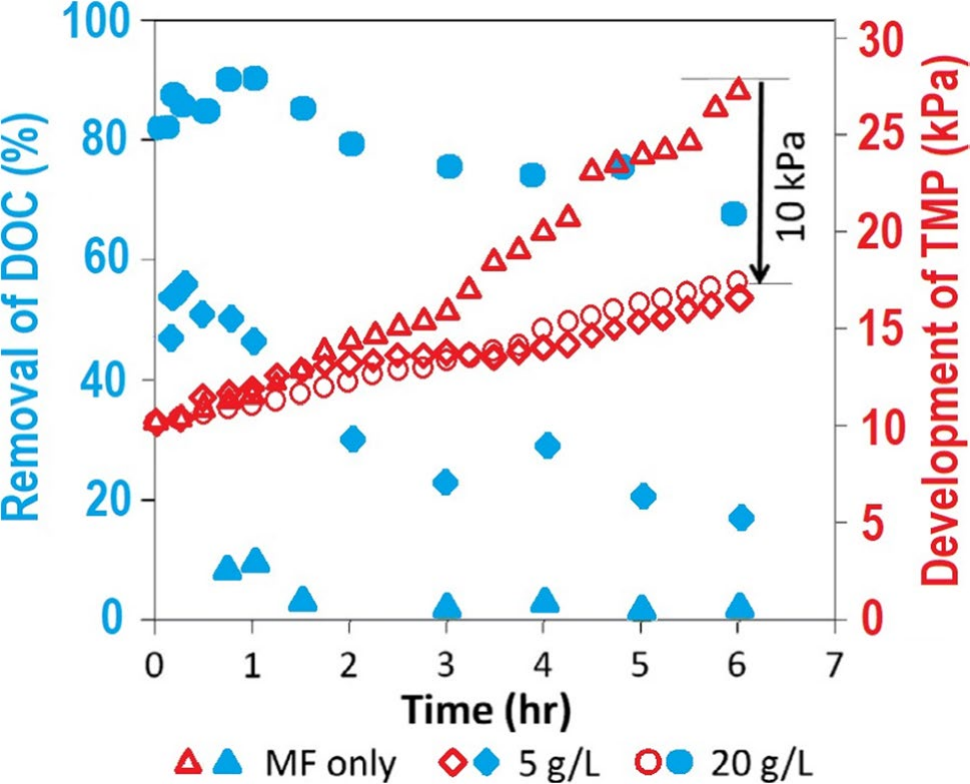
Removal of organics and TMP development in SMAHS (DOC of ROC 22–32 mg/L; flux 36 L/m.^2^ h; GAC doses 5 g/L and 20 g/L) (modified from Shanmuganathan et al. (2015b))

Similar to wastewater treatment, increasing the dose of GAC in MF/GAC SMAHS treatment of ROC sourced from a water reclamation plant raised the percentage removal of DOC and reduced the level of TMP development (Shanmuganathan et al. 2015b) (Fig. 5). MF filtration alone was not effective in removing DOC and was at less than 10%. This was because much of the organics were smaller than the MF membrane pore size of 0.1 µm, through which they passed. The TMP in the MF only system increased up to 27 kPa over the 6 h of the experiment. If 5 g/L of GAC was added at the start of the experiment, TMP development reduced by 10 kPa. Shanmuganathan et al. (2015b) reported that the smaller TMP development produced by 5 g/L of GAC was likely due to GAC pre-adsorbing the organics before it reached the membrane and the mechanical scouring of GAC on the surface of the membrane as it circulated around the reactor. Increasing the dose of GAC to 20 g/L did not further reduce TMP because the lower dose might have been sufficient to reduce fouling on the membrane. The result is unlikely the same with continuous operation of SMAHS over long duration experiments as others have found that higher dosages were required (Johir et al. 2011). Shanmuganathan et al. (2015b) demonstrated the important benefits of using SMAHS instead of MF alone for the treatment of ROC.

In the MF/GAC-based SMAHS treatment of the ROC, Shanmuganathan et al. (2015b) reported that the removal of the hydrophobic and hydrophilic fractions of DOC was nearly equal. Hydrophilic fraction of ROC contained mainly humics (500–1200 g/mol) and building blocks (weathered products of humics, 350–500 g/mol). SMAHS removed 37% and 69% of these fractions, respectively. Building blocks were smaller in size and could penetrate the pores of the GAC and become adsorbed in the internal surfaces resulting in higher percentage removal (Velten et al. 2011). The MF-GAC system with a GAC dose of 5 g/L effectively removed 17 OMPs by 65–100% from the ROC. A larger GAC dose of 20 g/L increased removal to 81–100%. There were no apparent relationships between OMP properties (molecular size, hydrophobicity and charge) and the observed removal efficiency. The authors claim that this was probably due to the high GAC dose used.

A later study of MF/GAC SMAHS treatment of ROC, using an initial 10 g/L GAC dose supplemented with a daily 10% GAC replacement, reported that DOC removal decreased from 80% on the first day to 50–60% after 4–6 days (Shanmuganathan et al. 2017). The authors explained that initially, DOC constituents with high affinity were adsorbed to GAC leaving in solution the lower affinity constituents. In time, this reversed as the low affinity DOC constituents increased in concentration at a higher rate than the high affinity constituents. By contrast, the removal of a majority of OMPs increased with time. The reason for this trend was that initially the OMPs were unable to compete strongly with high affinity DOC constituents for adsorption. As time progressed and as low affinity DOC constituents accumulated in solution, the OMPs were able to better compete for adsorption. In day 1, 60% and 76% hydrophilic or less hydrophobic DEET and sulfamethoxazole, respectively were removed. Other micropollutants were removed by > 81%. The removal of DEET, sulfamethoxazole and other micropollutants at day 7 was > 81–99%.

The cost-effectiveness of SMAHS is attractive both in terms of capital and the operational expenditures. Capital cost: In SMAHS, both the adsorption and membrane operations occur in a single tank, which reduces the cost. The prior adsorption helps to increase the permeate flux, and so reduces the required membrane area. Operational cost: Air scour rate is reduced due to the mechanical scour created by the suspended medium. This reduces the energy requirement and the operational cost. The mechanical scour and prior organic removal by the adsorbent reduce organic fouling on the membrane avoiding the expensive frequent chemical cleaning operation of the membrane. Periodic replacement of adsorbent into the reactor (2–3% of the adsorbent amount in the reactor) significantly reduces the required total amount of the adsorbent. For example, in SMAHS, a small amount of PAC (approximately 10 g/m^3^ of water treated) is sufficient to obtain an effluent with low organic/bio-fouling potential.

## Conclusions

Submerged membrane bioreactor systems can remove general organic pollutants, micro-organisms and solids. However, their capability in removing OMP including emerging contaminants of concern is not guaranteed. Combining the adsorption and membrane processes together means the advantages of both processes are realised and promise superior product water quality and improved process stability. Its success is mainly due to the following:

1. Optimization of backwash: For successful long-term operation of the membrane process, it is necessary to optimise the frequency and duration of the backwash. Periodic backwash duration and frequency use too large a volume of permeate for backwash, reducing the productivity of the system and raising the energy requirement. Adaptive backwash initiation and duration schemes with new control systems can lead to 40–50% reduction in backwash water and energy consumption.
2. Incorporation of adsorbent in SMAHS: The adsorbent produces an additional shearing effect that scours the membrane surface reducing particle deposition and lowers membrane resistance. It directly removes organics that would otherwise deposit on the membrane and cause fouling. A large amount of adsorbent is added only at the beginning of the process (e.g. 5–10 g/L of AC) followed by a periodic daily substitution of as little as 2–5% of adsorbent equivalent to an average adsorbent residence time of 20–50 days in the tank. This helps to economise the use of adsorbent without it becoming exhausted.

The main adsorbents (AC based) typically used in SMAHS are hydrophobic and possess negative surface charges, and so effectively remove the hydrophobic and positively charged constituents of NOM and positively charged OMP. In this case, the removal of the hydrophilic constituents of NOM and negatively charged OMPs is not effective. Other adsorbents such as Purolites that possess positive surface charges and hydrophilic characteristics are used to increase the effectiveness of SMAHS with respect to these constituents. Special adsorbents are used for removing phosphates (iron oxide adsorbents) and recovering valuable metals.

Several laboratory and pilot studies have demonstrated that SMAHS is an attractive and cost-effective solution in water reclamation. However, there are some areas where more research is warranted. Alternative adsorbents or combination of adsorbents need to be explored to remove NOM and emerging contaminants of concern such as microplastics, per- and poly-fluoroalkyl substances, bis-phenol, disinfectant by-products and pharmaceutical and personal care products. Long-term adverse effect of membrane damage by mechanical scouring caused by adsorbents needs to be studied in detail. Detailed study is required on the use of adsorbents in SMAHS as bio-sorbents to reduce its amount (and thus the cost). This also reduces the amount of exhausted adsorbent slurry to be handled/disposed.

## Data Availability

Data used in this paper will be made available on request. There is no new data created in this study. This paper is a mini-review. All relevant references have been cited.
